# Prehospital norepinephrine administration reduces 30-day mortality among septic shock patients

**DOI:** 10.1186/s12879-022-07337-y

**Published:** 2022-04-06

**Authors:** Romain Jouffroy, Adèle Hajjar, Basile Gilbert, Jean Pierre Tourtier, Emmanuel Bloch-Laine, Patrick Ecollan, Josiane Boularan, Vincent Bounes, Benoit Vivien, Papa-Ngalgou Gueye

**Affiliations:** 1 Intensive Care Unit, Ambroise Paré Hospital, Assistance Publique Hôpitaux Paris and Paris Saclay University, 9 avenue Charles de Gaulle, 92100 Boulogne-Billancourt, France; 2 Intensive Care Unit, Anaesthesiology, SAMU, Necker Enfants Malades Hospital, Assistance Publique, Hôpitaux Paris, Paris, France; 3 Centre de Recherche en Epidémiologie et Santé des Populations, U1018 INSERM, Paris Saclay University, Paris, France; 4 Institut de Recherche bioMédicale et d’Epidémiologie du Sport, EA7329, INSEP, Paris University, Paris, France; 5 EA 7525 Université des Antilles, Pointe-à-Pitre, France; 6 Department of Emergency Medicine, SAMU 31, University Hospital of Toulouse, Toulouse, France; 7 Paris Fire Brigade, Paris, France; 8 Emergency Department, Cochin Hospital, Paris, France; 9 Intensive Care Unit, SMUR, Pitie Salpêtriere Hospital, Assistance Publique, Hôpitaux Paris, 47 Boulevard de l’Hôpital, 75013 Paris, France; 10 SAMU 31, Centre Hospitalier Intercommunal Castres-Mazamet, Castres, France; 11 SAMU 972 University Hospital of Martinique, Fort-de-France, Martinique France; 12 Emergency Department, SMUR, Hôtel Dieu Hospital, Assistance Publique, Hôpitaux Paris, Paris, France

**Keywords:** Septic shock, Early, Prehospital setting, Norepinephrine, Mortality

## Abstract

**Background:**

Despite differences in time of sepsis recognition, recent studies support that early initiation of norepinephrine in patients with septic shock (SS) improves outcome without an increase in adverse effects. This study aims to investigate the relationship between 30-day mortality in patients with SS and prehospital norepinephrine infusion in order to reach a mean blood pressure (MAP) > 65 mmHg at the end of the prehospital stage.

**Methods:**

From April 06th, 2016 to December 31th, 2020, patients with SS requiring prehospital Mobile Intensive Care Unit intervention (MICU) were retrospectively analysed. To consider cofounders, the propensity score method was used to assess the relationship between prehospital norepinephrine administration in order to reach a MAP > 65 mmHg at the end of the prehospital stage and 30-day mortality.

**Results:**

Four hundred and seventy-eight patients were retrospectively analysed, among which 309 patients (65%) were male. The mean age was 69 ± 15 years. Pulmonary, digestive, and urinary infections were suspected among 44%, 24% and 17% patients, respectively. One third of patients (n = 143) received prehospital norepinephrine administration with a median dose of 1.0 [0.5–2.0] mg h^−1^, among which 84 (69%) were alive and 38 (31%) were deceased on day 30 after hospital-admission. 30-day overall mortality was 30%. Cox regression analysis after the propensity score showed a significant association between prehospital norepinephrine administration and 30-day mortality, with an adjusted hazard ratio of 0.42 [0.25–0.70], p < 10^–3^. Multivariate logistic regression of IPTW retrieved a significant decrease of 30-day mortality among the prehospital norepinephrine group: ORa = 0.75 [0.70–0.79], p < 10^–3^.

**Conclusion:**

In this study, we report that prehospital norepinephrine infusion in order to reach a MAP > 65 mmHg at the end of the prehospital stage is associated with a decrease in 30-day mortality in patients with SS cared for by a MICU in the prehospital setting. Further prospective studies are needed to confirm that very early norepinephrine infusion decreases septic shock mortality.

## Background

Annually, sepsis affects more than 30 million people worldwide [1–3]. Sepsis represents one of the leading causes of morbidity and mortality among patients admitted to the intensive care unit (ICU) [4] with an overall mortality rate varying between 25 and 50% [5–7]. Every year, all over the world, around 11 million deaths are related to sepsis [3], among which one-third to one-half occur in-hospital [8].

From a pathophysiological point of view, sepsis leads to an absolute and relative hypovolemia, due to a vascular tone decrease, reflected by micro, e.g., skin mottling, and macrocirculation, e.g., hypotension, parameters alteration. Consequently, in order to correct hypovolemia, to restore the vascular tone, and to ensure tissues perfusion [9, 10], the guidelines recommend early fluid volume expansion, at least 30 ml kg^−1^ of intravenous crystalloids within the first 3 h, and norepinephrine infusion [11–13]. However, excessive fluid resuscitation, i.e., undue fluid volume expansion, results in a risk of fluid overload [14], and is independently associated with increased mortality in septic shock [15–19]. Otherwise, recent experimental data report a paradoxical increase in vasopressor requirement after high fluid volume expansion secondary to endothelial damage related to atrial natriuretic peptide shedding [20].

Consequently, the optimal timing to start norepinephrine administration is a question of utmost importance. Recent guidelines updates recommend early, within a “1-h bundle” of, norepinephrine administration, before fluid volume expansion achievement/failure should be performed in order to maintain a mean arterial pressure of at least 65 mmHg [21, 22]. However, for in-hospital patients, T0 time for “1-h bundle” initiation varies between studies due to the diagnostic difficulties of initial sepsis severity. To date, most cases of sepsis (70%) occur outside hospital environment [23], and the median time to hospital transport is around 1 h. Prompt prehospital correction of hypotension improves septic shock survival [24] according to previous in-hospital studies reporting the influence of delays in correcting hypotension [25–27]. A recent systematic review and meta-analysis among in-hospitalized septic shock reported that early norepinephrine administration in patient with septic shock was associated with decreased short-term mortality, a shorter time to achieved target mean arterial pressure, and lower fluid expansion within 6 h [28].

Nevertheless, the impact of very early norepinephrine administration, since the prehospital setting, in patients with septic shock, remains unexplored. This study aims to investigate the relationship between prehospital norepinephrine administration in order to reach a MAP > 65 mmHg at the end of the prehospital stage and 30-day mortality in patients with septic shock.

## Methods

### Patients

As previously reported [29], in France, a public health control organisation, the SAMU (Urgent Medical Aid Service) provides the medical response to prehospital emergency medical situations. The SAMU is a dispatch centre where a team of assistants and physicians answer calls and triage the patients’ complaints [30]. SAMU may send to the scene the SMUR (Mobile Emergency and Resuscitation Service), a mobile intensive care unit (MICU), in order to provide out-of-hospital care and to transport to a definitive in-hospital facility: either the emergency department (ED) or directly to the intensive care unit (ICU). For life-threatening emergencies, the MICU team is able to manage major emergencies [31]. The MICU team is composed of a driver, a nurse and an emergency physician [31].

From April 06th, 2016 to December 31th, 2020, patients with a diagnosis of septic shock according to the 2012 sepsis-2 conference [32] cared for by a MICU teams of one of 7 French hospital centres (Necker-Enfants malades Hospital, Lariboisière Hospital, La Pitié-Salpêtrière Hospital, Hôtel Dieu Hospital, APHP, Paris—France; The Paris Fire Brigade Paris,—France; The Toulouse University Health Centre, Toulouse—France and the Castres Hospital, Castres—France) were retrospectively included and patients care records were retrospectively analyzed. Patients who were younger than 18 years, and/or are pregnant, and/or have serious comorbid conditions with an unknown prehospital life support and/or with guardianship or curatorship were not included. Treatments management and strategy used to achieve an MAP at the end of prehospital care were left to the MICU physician’s discretion.

Patients’ demographic characteristics (age, weight, height, and gender), suspected prehospital origin of sepsis, initial prehospital (e.g., the first MICU contact), and final prehospital (e.g., at the end of prehospital stage) vital sign values [systolic (SAP), diastolic (DAP) and mean arterial pressure (MAP)] were measured with a non-invasive automated device in all centres. Heart rate (HR), pulse oximetry (SpO_2_), respiratory rate (RR), temperature and Glasgow coma scale (GCS), plasma blood glucose, duration of prehospital care, and prehospital treatments delivered (antibiotic therapy type and dose, fluid volume expansion type and dose, as well as catecholamine type and dose) were collected from MICU prehospital medical reports. Determination of volume per kg of prehospital fluid resuscitation was based on the recorded ideal body weight.

Previous underlying comorbidities (chronic cardiac failure, chronic renal failure, chronic obstructive pulmonary disease, diabetes mellitus, and history of cancer) were also collected in order to take into account the underlying condition [33].

The length of stay (LOS) in the ICU, in-hospital LOS, and the 30-day mortality were retrieved from medical reports in case of in-hospital death or by call when the patient was discharged from the hospital. The Sequential Organ Failure Assessment (SOFA) score [34] and the Simplified Acute Physiology Score (SAPS 2) [35] were calculated 24 h after ICU admission.

### Ethical considerations

The study was approved by the French Society of Anaesthesia and Intensive Care ethics committee on December 12th, 2017 (Ref number: IRB 00010254-2017-026). The ethics committee considered that consent of patients was waived for participation in this observational study.

### Statistical analysis

Results are expressed as a mean with standard deviation for quantitative parameters with a normal distribution, as median with interquartile range [Q1–Q3] for parameters with a non-Gaussian distribution, and as absolute values and percentages for qualitative parameters.

The primary outcome was the 30-day mortality rate. The secondary outcomes were the in-ICU LOS and in-hospital LOS.

Univariate and multivariate analyses were performed to evaluate the relationship between each covariate and the 30-day mortality rate.

Prehospital norepinephrine administration was encoded as a qualitative covariate (0 = absent and 1 = present), for norepinephrine infusion in order to reach a MAP > 65 mmHg at the end of the prehospital stage.

In order to reduce the effect of potential confounders on primary and secondary outcomes, a propensity score matching was used to balance the differences in baseline characteristics between patients with prehospital norepinephrine administration and those without prehospital norepinephrine administration. The propensity score (i.e., the probability of prehospital norepinephrine administration), was estimated using logistic regression based on potential confounders on 30-day mortality: age, chronic cardiac failure, SAPS2 score, bacteriological identification, chronic obstructive pulmonary disease, initial mean blood pressure, i.e., the first measurement performed in the prehospital setting, history of cancer, prehospital fluid expansion, prehospital antibiotic administration, prehospital duration and MICU centre.

The nearest neighbour matching method was used to match patients based on the logit of the propensity score. The balance of covariates after matching was assessed by absolute mean differences with a considered acceptable threshold of 10%. A survival analysis using Cox proportional hazards regression was used to compare the 30-day mortality of patients with and without prehospital norepinephrine administration in the propensity score–matched cohort. Proportional hazards assumption was verified for each Cox model variable by Kaplan Meier curves and the log-rank test. To eliminate the potential bias related to the potential interaction between prehospital AB administration and prehospital norepinephrine administration, we performed a survival analysis using Cox proportional hazards regression including an interaction term between prehospital AB administration and prehospital norepinephrine administration.

Additional sensitivity analyses were performed on different final prehospital MAP levels (> 70, 75 and 80 mmHg) independent of a pre-existing hypertension history and in 2 subgroups: (1) MAP > 65 mmHg if no hypertension history and (2) MAP > 75 mmHg if pre-existing hypertension. To assess the causal link between 30-day mortality rate and prehospital norepinephrine administration, an Inverse Probability Treatment Weighting (IPTW) method without weight truncation was used to control for confounding on the crude cohort.

Results are expressed by an adjusted Hazard ratio (aHR) with 95 percent confidence intervals [95 CI]. All tests were 2-sided with a statistically significant *p-value* of < 0 0.05. All analyses were performed using R 3.4.2 (http://www.R-project.org; the R Foundation for Statistical Computing, Vienna, Austria).

## Results

### Patient characteristics

Four hundred seventy-eight patients with septic shock requiring action by a prehospital MICU were included in this study. Among them, 309 patients (65%) were male, and the mean age was 69 ± 15 years old (Table 1).

**Table 1:** Population characteristics

	Overall population (n = 478)	Living (n = 332)	Deceased (n = 146)	p value
Demographics				
Age (years)	69 ± 15	67 ± 15	73 ± 14	**2.10**^**–3**^
Hypertension	196 (41%)	134 (40%)	62 (42%)	0.937
Chronic cardiac failure	105 (22%)	52 (16%)	53 (36%)	**< 10**^**–3**^
Diabetes mellitus	129 (27%)	94 (28%)	35 (24%)	0.120
Cancer history	169 (35%)	104 (31%)	65 (44%)	**0.011**
COPD	70 (15%)	45 (14%)	25 (17%)	0.203
Chronic renal failure	68 (14%)	41 (12%)	27 (18%)	0.082
Prehospital				
SBP (mmHg)	98 ± 41	100 ± 46	94 ± 29	0.095
DBP (mmHg)	58 ± 20	59 ± 21	55 ± 20	0.058
MAP (mmHg)	70 ± 22	72 ± 23	68 ± 22	0.112
HR (beats min^−1^)	114 ± 29	115 ± 27	112 ± 30	0.375
RR (movements min^−1^)	30 [22–36]	28 [31–35]	31 [25–38]	**0.043**
Pulse oximetry (%)	92 [85–96]	93 [86–97]	90 [83–95]	**0.021**
Body core temperature (°C)	38.3 [36.4–39.1]	38.5 [36.8–39.3]	38.0 [35.8–39.0]	**0.007**
Glasgow coma scale	15 [13–15]	15 [13–15]	14 [11–15]	**0.010**
Blood lactate (mmol l^−1^)	5.9 ± 3.4	5.8 ± 3.4	6.3 ± 3.6	0.313
Fluid expansion (ml)	750 [500–100]	750 [500–1000]	750 [500–1200]	0.464
Fluid expansion indexed on body weight (ml kg^−1^)	14 ± 9	14 ± 9	14 ± 9	0.890
Norepinephrine administration	143 (30%)	99 (30%)	44 (30%)	0.927
Norepinephrine dose (mg h^−1^)	1.0 [0.5–2.0]	1.0 [0.5–2.0]	1.3 [1.0–2.0]	0.065
Prehospital AB administration	124 (26%)	91 (72%)	33 (23%)	0.206
Prehospital duration (min)	72 ± 34	70 ± 34	74 ± 34	0.291
Hospital				
Initial SBP (mmHg)	105 ± 26	106 ± 26	101 ± 26	**0.016**
Initial DBP (mmHg)	62 ± 19	63 ± 19	59 ± 19	**0.040**
Initial MBP (mmHg)	76 ± 20	77 ± 19	73 ± 20	**0.023**
Initial HR (beats min^−1^)	107 ± 26	107 ± 24	106 ± 29	0.732
Initial RR (movements min^−1^)	25 [19–30]	24 [18–30]	26 [20–35]	**0.011**
Initial pulse oximetry (%)	97 [94–99]	97 [95–99]	97 [93–98]	**10**^**–3**^
Initial body core temperature (°C)	38.0 [36.0–39.0]	39.0 [37.0–39.0]	36.5 [35.0–38.8]	**0.009**
Initial Glasgow coma scale	15 [14–15]	15 [14–15]	14 [12–15]	**< 10**^**–3**^
Initial blood lactate (mmol l^−1^)	4.3 ± 3.4	3.6 ± 2.9	5.8 ± 3.9	**< 10**^**–3**^
SOFA score	6 [4–10]	6 [3–9]	8 [5–11]	**< 10**^**–3**^
SAPS2 score	61 ± 22	55 ± 20	72 ± 21	**< 10**^**–3**^
In-ICU length of stay (days)	4 [2–8]	5 [2–9]	3 [1–8]	**0.009**
In-hospital length of stay (days)	10 [5–18]	14 [8–23]	5 [2–11]	**< 10**^**–3**^

Results were expressed as mean and standard deviation for quantitative parameters (normal distribution), as median and interquartile range for quantitative parameters (non-gaussian distribution), and as absolute value and percentage for qualitative parameters. P-value corresponds to the comparison between deceased and living patients

*SBP* systolic blood pressure, *DBP* diastolic blood pressure, *MBP* mean blood pressure, *HR* heart rate, *RR* respiratory rate, *ICU* intensive care unit, *SOFA* sequential organ failure assessment, *SAPS2* simplified acute physiology score 2nd version, *HIV* human immunodeficiency virus, *COPD* chronic obstructive pulmonary disease, *AB* antibiotic therapy, *min* minutes

Values in bold indicate a p-value < 0.05 between living and deceased patients

Pulmonary, digestive and urinary infections were suspected among 44%, 24% and 17% patients, respectively (Table 2).

**Table 2:** Presumed septic shock origins

Origin	n (percentage)
Pulmonary	210 (44%)
Digestive	115 (24%)
Urinary	80 (17%)
Cutaneous	30 (6%)
Meningeal	9 (2%)
Gynaecological	3 (1%)
Ears nose throat	2 (0.5%)
Cardiovascular	2 (0.5%)
Unknown	27 (6%)

Data are expressed in absolute value and the corresponding percentages are indicated into brackets. Due to percentage rounding, the sum overpasses 100%

No significant difference in the prehospital stage duration was observed between patients who survived and those who died (Table 1).

No significant difference in the prehospital fluid expansion indexed on real body weight was observed between survival and deceased patients (Table 1).

No significant difference in terms of survival was observed between patients who received and those who did not receive prehospital antibiotic therapy (Table 1).

Among the 124 patients (26%) who received antibiotics prior to hospital admission, 74% were treated with 3rd generation cephalosporin among which 59% was with cefotaxime and 61% with ceftriaxone.

The median ICU length of stay was 4 [2–8] days and the median length of stay in a hospital was 10 [5–18] days (Table 1).

The 30-day overall mortality rate reached 31%.

### Primary outcome

One hundred and forty-three patients (30%) received prehospital norepinephrine administration with a median dose of 0.25 [0.13–0.47] mcg kg^−1^ min^−1^. Among them, 99 patients (69%) were alive whereas 44 (30%) were deceased on day 30 after hospital-admission (p = 0.927) (Table 3).

**Table 3:** Characteristics of patients with prehospital norepinephrine administration (early NE) and patients without prehospital norepinephrine administration (non early NE)

	Non early NE (n = 335)	Early NE (n = 143)	p value
Demographics			
Age (years)	**70 ± 16**	**67 ± 12**	**0.066**
Hypertension	**145 (43%)**	**51 (36%)**	**0.122**
Chronic cardiac failure	**74 (22%)**	**31 (22%)**	**0.921**
Diabetes mellitus	**84 (25%)**	**45 (31%)**	**0.150**
Cancer history	**118 (35%)**	**51 (36%)**	**0.927**
COPD	**43 (13%)**	**27 (19%)**	**0.089**
Chronic renal failure	**53 (16%)**	**15 (10%)**	**0.129**
Prehospital			
SBP (mmHg)	**104 ± 45**	**84 ± 26**	** < 10**^**–3**^
DBP (mmHg)	**61 ± 19**	**50 ± 21**	** < 10**^**–3**^
MAP (mmHg)	**74 ± 21**	**61 ± 22**	**< 10**^**–3**^
HR (beats min^−1^)	**113 ± 28**	**116 ± 30**	**0.323**
RR (movements min^−1^)	**30 **[22–36]	**29 **[22–35]	**0.964**
Pulse oximetry (%)	**92 [85–96]**	**92 [84–96]**	**0.225**
Body core temperature (°C)	**38.4 [36.9–39.1]**	**38.0 [36.1–39.1]**	**0.078**
Glasgow coma scale	**15 **[13–15]	**14 **[10–15]	**< 10**^**–3**^
Blood lactate (mmol l^−1^)	**5.4 ± 3.1**	**6.7 ± 3.7**	**0.006**
Fluid expansion (ml)	**700 [500–1000]**	**1000 [750–1500]**	**< 10**^**–3**^
Fluid expansion indexed on body weight (ml kg^−1^)	**12 ± 8**	**18 ± 10**	**< 10**^**–3**^
Norepinephrine administration	**0 (0%)**	**143 (100%)**	**–**
Norepinephrine dose (mcg kg^−1^ min^−1^)	**0.25 [0.13–0.47]**	**–**	**–**
Prehospital AB administration	**92 (33%)**	**32 (22%)**	**0.206**
Prehospital duration (min)	**73 ± 29**	**80 ± 36**	**0.093**
Hospital			
Initial SBP (mmHg)	**105 ± 25**	**104 ± 27**	**0.761**
Initial DBP (mmHg)	**62 ± 18**	**61 ± 20**	**0.887**
Initial MBP (mmHg)	**76 ± 19**	**76 ± 22**	**0.891**
Initial HR (beats min^−1^)	**108 ± 25**	**104 ± 26**	**0.067**
Initial RR (movements min^−1^)	**25 [20–32]**	**22 [17–30]**	**0.002**
Initial pulse oximetry (%)	**97 [94–99]**	**97 [95–99]**	**0.303**
Initial body core temperature (°C)	**38.0 [36.0–39.0]**	**36.1 [35.8–39.1]**	**0.768**
Initial Glasgow coma scale	**15 [14–15]**	**14 [14–15]**	**0.890**
Initial blood lactate (mmol l^−1^)	**4.0 ± 3.1**	**5.1 ± 3.9**	**0.004**
SOFA score	**5 [3–9]**	**8 [5–10]**	**< 10**^**–3**^
SAPS2 score	**59 ± 20**	**63 ± 24**	**0.091**
In-ICU length of stay (days)	**3 [1–8]**	**6 [3–10]**	**0.023**
In-hospital length of stay (days)	**10 **[5–18]	**12 **[6–21]	**0.107**

Results were expressed as mean and standard deviation for quantitative parameters (normal distribution), as median and interquartile range for quantitative parameters (non-Gaussian distribution), and as absolute value and percentage for qualitative parameters. The p-value corresponds to the comparison between patients with prehospital norepinephrine administration (early NE) and patients without prehospital norepinephrine administration (non early NE)

*SBP* systolic blood pressure, *DBP* diastolic blood pressure, *MBP* mean blood pressure, *HR* heart rate, *RR* respiratory rate, *ICU* intensive care unit, *SOFA* sequential organ failure assessment, *SAPS2* simplified acute physiology score 2nd version, *HIV* human immunodeficiency virus, *COPD* chronic obstructive pulmonary disease, *AB* antibiotic therapy, *min* minutes

Values in bold indicate a p-value < 0.05 between living and deceased patients

Bivariate analysis reported a significant association between 30-day mortality and age, chronic cardiac failure, cancer, prehospital initial body core temperature, initial Glasgow coma scale, initial respiratory rate, initial pulse oximetry, final systolic blood pressure, final diastolic pressure, final mean arterial pressure, final respiratory rate, final pulse oximetry, final Glasgow coma scale, final body core temperature, final blood lactatemia, in ICU and in-hospital length of stay, SOFA score and SAPS2 score (Table 1).

### Survival analysis

**Fig. 1:**
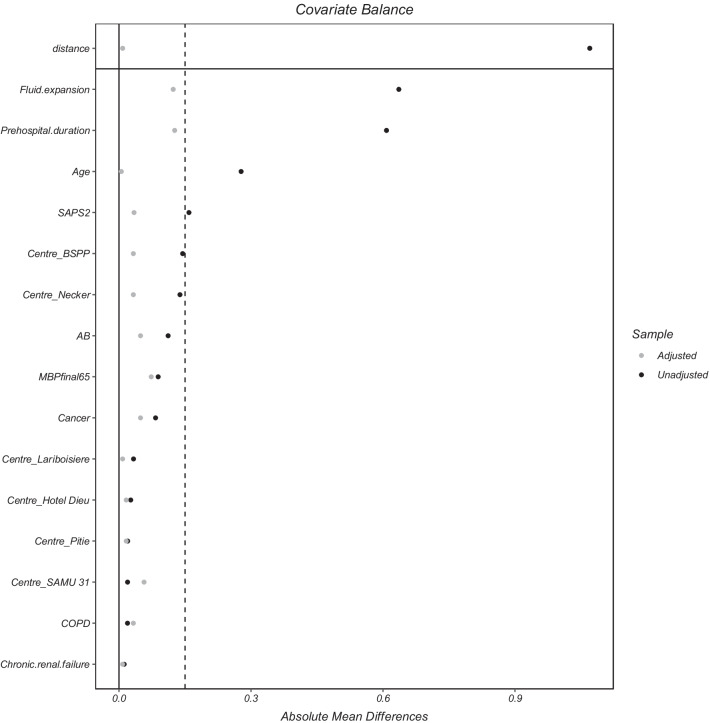
Absolute mean differences between patients with prehospital norepinephrine optimisation and those without prehospital norepinephrine optimisation achievement after matching

The matched population consists of 52 controls, i.e., no prehospital norepinephrine administration and 123 cases, i.e., prehospital norepinephrine administration. The absolute mean differences between cases and controls after propensity score matching are depicted in Fig. 1.

**Fig. 2:**
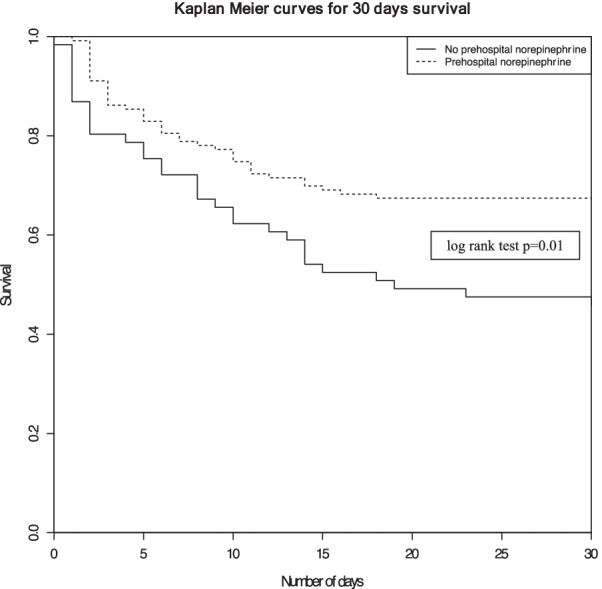
Kaplan Meier curves for 30-days survival between patients with prehospital norepinephrine administration and those without prehospital norepinephrine administration

Using Cox regression analysis after matching, prehospital norepinephrine administration was significantly associated with a 30-day mortality decrease, an adjusted hazard ratio (aHR) of 0.42 [0.25–0.70], and a log rank test p = 0.01. Kaplan Meier curves depict differences on 30-day survival in both subgroups after adjustment of confounders (Fig. 2).

Cox regression analysis including an interaction term between prehospital AB administration and prehospital norepinephrine administration, reported a non-significant association with 30-day mortality (aHR: 1.11 [0.39–3.19], p = 0.85).

### Sensitivity analysis

Using 3 different levels for final prehospital MAP (> 70, 75 and 80 mmHg) independent of a pre-existing hypertension history, Cox regression analysis did not result in an increase of significance between prehospital norepinephrine administration and 30-day mortality (aHR = 0.50 [0.25–0.70], aHR = 0.48 [0.26–0.87], aHR = 0.57 [0.34–0.97] respectively, p = 0.01). Conversely, in the 2 subgroups based on previous hypertension history, Cox regression analysis results showed a persistent significant association between prehospital norepinephrine administration and 30-day mortality: aHR = 0.42 [0.24–0.75], p < 10^–3^.

In a multivariate logistic regression model of Inverse Probability Treatment Weighting, the 30-day mortality in the prehospital norepinephrine group significantly decreased (aOR = 0.75 [0.70–0.79], p < 10^–3^).

## Discussion

In this study, we observed that prehospital norepinephrine infusion in order to reach a MAP of at least 65 mmHg in patients with septic shock cared for by a MICU in the prehospital setting is associated with a decrease in 30-day mortality.

Sepsis leads to a systemic response inflammatory syndrome (SRIS) characterised by an absolute hypovolemia related to vascular leakage and by a relative hypovolemia related to the systemic vasodilatation, both reflected by macro circulatory (e.g., low blood pressure) and microcirculatory (e.g., blood lactate or skin mottling) markers [36]. Previous studies reported that the association between these clinical and biological microcirculatory failure parameters are associated with a poor outcome [37, 38].

In order to restore the organs’ perfusion, i.e., by restoring cardiac output and mean arterial pressure, and the tissue perfusion surrogate, the 2016 SEPSIS-3 conference recommends early fluid expansion (30 ml kg^−1^ of intravenous crystalloid within 3 h after sepsis recognition) and catecholamine, norepinephrine infusion, when MAP remains lower than 65 mmHg [12]. Moreover, the Surviving Sepsis Campaign 2019 recommendations updates, even advocate, the use of vasopressors during or after fluid resuscitation to reach and maintain a MAP ≥ 65 mmHg within the first hour after sepsis recognition [22]. Our results are in line with previous studies reporting evidence that uncontrolled and excessive fluid resuscitation is not safe [13, 16]. Several observational studies have reported an association between sepsis mortality, (1) the volume of resuscitation fluids and (2) net fluid balance [14–19]. In order to avoid fluid overload consequences [15–19, 39], recent data support that norepinephrine infusion in combination with, but not without [40], fluid resuscitation is feasible without increasing adverse effects [41]. Moreover, norepinephrine should be started as soon as possible without expecting fluid resuscitation failure in order to prevent organ(s) dysfunction as observed for acute kidney injury directly influenced by both severity and duration of hypotension [42–44]. Beyond this, acute kidney injury prevention, and early norepinephrine administration is also associated with a mortality decrease [28, 45–48]. The beneficial effects of norepinephrine are mediated by the cardiac output increase related to the cardiac preload and contractility increases, and an improvement in microcirculation [45]. A strength of our study relies on the fact that norepinephrine administration is started (1) within the first hour after septic shock diagnosis and (2) before the end of the fluid resuscitation according to the recent Surviving Sepsis Campaign 2019 recommendations updates [22] without any adverse effects. Moreover, contrary to previous studies where the T0 time for “1-h bundle” initiation suffers from variability, in our study the T0 time is the same for every patient, even if the delays from sepsis occurrence varies among patients.

The SEPSIS-3 conference [12] and the Surviving Sepsis Campaign [11] recommend a MAP target of at least 65 mmHg. Nevertheless, a unique MAP target, without taking into account the underlying cardiovascular condition, especially hypertension history, does not appear adequate from a physiological point of view. Chronic hypertension induces physiological changes (e.g., the right shift of brain autoregulation) [26, 49, 50] and previous studies reported benefits of reaching a higher MAP on cardiac function [51–53], on microcirculation [54], and on renal function [43]. Recently, Lee et al. reported a better prognosis with a MAP target between 75 and 85 mmHg [55], which we also observed in the subgroup of patients with hypertension history. Consequently, we believe that hypertension history should be taken into account even at the very early stage of septic shock resuscitation.

Contrary to previous results [29], the lack of a significance in survival between prehospital antibiotic therapy and no prehospital antibiotic therapy may be explained by the relative higher weight of hemodynamic achievement vs early antibiotic therapy. If early appropriate treatments implementation, especially antibiotic therapy and hemodynamic optimization [12, 29, 56–61], is crucial, a sufficient tissue perfusion pressure achievement is all the more important to allow the antibiotic therapy to reach infected tissues [62, 63].

This study presents some limitations that should be considered.

This is a retrospective study; therefore, we cannot conclude that physicians prescribed norepinephrine administration after or before fluid expansion failure nor for a specific mean arterial pressure target. Data were collected from prehospital and ICU-medical reports leading to the possibility of a misclassification bias. The generalization of the results obtained from adult septic shock patients is not transposable to paediatric patients. The statistical analysis allows only conclusion for a link between 30-day mortality and prehospital norepinephrine administration, not a causality link. We do not report the incidence of adverse events related to the peripheral norepinephrine infusion. The specificity of the French prehospital emergency medical service based on MICU affects the results’ external validity. However, the herein study results suggest that (1) the treatments instituted in the prehospital setting affect the outcome more than the EMS organisation, and (2) that taking into account underlying hypertension history is important.

Beyond all the previous considerations, our study strength is that we considered the sicker septic patients, i.e., those with septic shock, for whom hemodynamic restauration cannot suffer from any delay. Nevertheless, our results must be confirmed by larger prospective studies in order to confirm the real impact of very early norepinephrine administration, considering the possibility of an underlying hypertension.

## Conclusion

Prehospital norepinephrine infusion in order to reach a mean arterial pressure > 65 mmHg is associated with a decrease in 30-day mortality in patients with septic shock cared for by a mobile intensive care unit in the prehospital setting. Previous hypertension history should be considered from the prehospital stage of septic shock resuscitation to determine the optimal mean arterial pressure target. Prospective studies are needed to confirm that very early norepinephrine infusion allow to increase septic shock survival.

## Data Availability

Data and material are available on reasonable request.

## Abbreviations

MICU: Mobile Intensive Care Unit intervention
ICU: Intensive Care Unit intervention
MAP: Mean arterial pressure
SS: Septic shock
SAMU: Urgent medical aid service
SMUR: Mobile emergency and resuscitation service
ED: Emergency department
SFAR: French Society of Anaesthesia and Intensive Care
SRLF: French Society of Intensive Care
SAP: Systolic arterial pressure
DAP: Diastolic arterial pressure
HR: Heart rate
SpO_2_: Pulse oximetry
RR: Respiratory rate
GCS: Glasgow coma scale
LOS: Length of stay
SOFA: Sequential organ failure assessment
SAPS 2: Simplified acute physiology score
IPTW: Inverse probability treatment weighting
ORa: Adjusted odds ratio
aHR: Adjusted Hazard ratio

## References

[1] Fleischmann C, Scherag A, Adhikari NK, Hartog CS, Tsaganos T, Schlattmann P (2016). Assessment of global incidence and mortality of hospital-treated. Sepsis current estimates and limitations. Am J Respir Crit Care Med.

[2] Gaieski DF, Edwards JM, Kallan MJ, Carr BG (2013). Benchmarking the incidence and mortality of severe sepsis in the United States. Crit Care Med.

[3] Rudd KE, Johnson SC, Agesa KM, Shackelford KA, Tsoi D, Kievlan DR (2020). Global, regional, and national sepsis incidence and mortality, 1990–2017: analysis for the Global Burden of Disease Study. Lancet.

[4] Vincent JL, Jones G, David S, Olariu E, Cadwell KK (2019). Frequency and mortality of septic shock in Europe and North America: a systematic review and meta-analysis. Crit Care.

[5] Martin GS, Mannino DM, Eaton S, Moss M (2003). The epidemiology of sepsis in the United States from 1979 through 2000. N Engl J Med.

[6] Ranieri VM, Thompson BT, Barie PS, Dhainaut JF, Douglas IS, Finfer S (2012). Drotrecogin alfa (activated) in adults with septic shock. N Engl J Med.

[7] Kumar A, Ellis P, Arabi Y, Roberts D, Light B, Parrillo JE (2009). Initiation of inappropriate antimicrobial therapy results in a fivefold reduction of survival in human septic shock. Chest.

[8] Liu V, Escobar GJ, Greene JD, Soule J, Whippy A, Angus DC (2014). Hospital deaths in patients with sepsis from 2 independent cohorts. JAMA.

[9] Levy MM, Evans LE, Rhodes A (2018). The surviving sepsis campaign bundle: 2018 update. Crit Care Med.

[10] De Backer D, Orbegozo Cortes D, Donadello K, Vincent JL (2014). Pathophysiology of microcirculatory dysfunction and the pathogenesis of septic shock. Virulence.

[11] Rhodes A, Evans LE, Alhazzani W, Levy MM, Antonelli M, Ferrer R (2017). Surviving sepsis campaign: international guidelines for management of sepsis and septic shock: 2016. Intensive Care Med.

[12] Singer M, Deutschman CS, Seymour CW, Shankar-Hari M, Annane D, Bauer M (2016). The Third International Consensus Definitions for Sepsis and Septic Shock (Sepsis-3). JAMA.

[13] Levy MM, Evans LE, Rhodes A (2018). The surviving sepsis campaign bundle: 2018 update. Intensive Care Med.

[14] Kelm DJ, Perrin JT, Cartin-Ceba R, Gajic O, Schenck L, Kennedy CC (2015). Fluid overload in patients with severe sepsis and septic shock treated with early goal-directed therapy is associated with increased acute need for fluid-related medical interventions and hospital death. Shock.

[15] Boyd JH, Forbes J, Nakada TA, Walley KR, Russell JA (2011). Fluid resuscitation in septic shock: a positive fluid balance and elevated central venous pressure are associated with increased mortality. Crit Care Med.

[16] Marik PE, Malbrain M (2017). The SEP-1 quality mandate may be harmful: how to drown a patient with 30 mL per kg fluid!. Anaesthesiol Intensive Ther.

[17] Sakr Y, Rubatto Birri PN, Kotfis K, Nanchal R, Shah B, Kluge S (2017). Higher fluid balance increases the risk of death from sepsis: results from a large international audit. Crit Care Med.

[18] Sirvent JM, Ferri C, Baro A, Murcia C, Lorencio C (2015). Fluid balance in sepsis and septic shock as a determining factor of mortality. Am J Emerg Med.

[19] Silversides JA, Major E, Ferguson AJ, Mann EE, McAuley DF, Marshall JC (2017). Conservative fluid management or deresuscitation for patients with sepsis or acute respiratory distress syndrome following the resuscitation phase of critical illness: a systematic review and meta-analysis. Intensive Care Med.

[20] Byrne L, Obonyo NG, Diab SD, Dunster KR, Passmore MR, Boon AC (2018). Unintended consequences: fluid resuscitation worsens shock in an ovine model of endotoxemia. Am J Respir Crit Care Med.

[21] Scheeren TWL, Bakker J, De Backer D, Annane D, Asfar P, Boerma EC (2019). Current use of vasopressors in septic shock. Ann Intensive Care.

[22] Chen AX, Simpson SQ, Pallin DJ (2019). Sepsis guidelines. N Engl J Med.

[23] Brun-Buisson C, Meshaka P, Pinton P, Vallet B, Group ES (2004). EPISEPSIS: a reappraisal of the epidemiology and outcome of severe sepsis in French intensive care units. Intensive Care Med.

[24] Jouffroy R, Gilbert B, Gueye PN, Tourtier JP, Bloch-Laine E, Ecollan P (2021). Prehospital hemodynamic optimisation is associated with a 30-day mortality decrease in patients with septic shock. Am J Emerg Med.

[25] Varpula M, Tallgren M, Saukkonen K, Voipio-Pulkki LM, Pettila V (2005). Hemodynamic variables related to outcome in septic shock. Intensive Care Med.

[26] Dunser MW, Takala J, Ulmer H, Mayr VD, Luckner G, Jochberger S (2009). Arterial blood pressure during early sepsis and outcome. Intensive Care Med.

[27] Maheshwari K, Nathanson BH, Munson SH, Khangulov V, Stevens M, Badani H (2018). The relationship between ICU hypotension and in-hospital mortality and morbidity in septic patients. Intensive Care Med.

[28] Li Y, Li H, Zhang D (2020). Timing of norepinephrine initiation in patients with septic shock: a systematic review and meta-analysis. Crit Care.

[29] Jouffroy R, Gilbert B, Tourtier JP, Bloch-Laine E, Ecollan P, Bounes V (2020). Impact of prehospital antibiotic therapy on septic shock mortality. Prehosp Emerg Care.

[30] Jouffroy R, Saade A, Muret A, Philippe P, Michaloux M, Carli P (2018). Fluid resuscitation in pre-hospital management of septic shock. Am J Emerg Med.

[31] Adnet F, Lapostolle F (2004). International EMS systems: France. Resuscitation.

[32] Dellinger RP, Levy MM, Rhodes A, Annane D, Gerlach H, Opal SM (2013). Surviving Sepsis Campaign: international guidelines for management of severe sepsis and septic shock, 2012. Intensive Care Med.

[33] Salvatore F (2020). The shift of the paradigm between ageing and diseases. Clin Chem Lab Med.

[34] Vincent JL, Moreno R, Takala J, Willatts S, De Mendonca A, Bruining H (1996). The SOFA (Sepsis-related Organ Failure Assessment) score to describe organ dysfunction/failure. On behalf of the Working Group on Sepsis-Related Problems of the European Society of Intensive Care Medicine. Intensive Care Med.

[35] Le Gall JR, Lemeshow S, Saulnier F (1993). A new Simplified Acute Physiology Score (SAPS II) based on a European/North American multicenter study. JAMA.

[36] Angus DC, van der Poll T (2013). Severe sepsis and septic shock. N Engl J Med.

[37] Ait-Oufella H, Bige N, Boelle PY, Pichereau C, Alves M, Bertinchamp R (2014). Capillary refill time exploration during septic shock. Intensive Care Med.

[38] Charlton M, Sims M, Coats T, Thompson JP (2017). The microcirculation and its measurement in sepsis. J Intensive Care Soc.

[39] Marik PE, Linde-Zwirble WT, Bittner EA, Sahatjian J, Hansell D (2017). Fluid administration in severe sepsis and septic shock, patterns and outcomes: an analysis of a large national database. Intensive Care Med.

[40] Sennoun N, Montemont C, Gibot S, Lacolley P, Levy B (2007). Comparative effects of early versus delayed use of norepinephrine in resuscitated endotoxic shock. Crit Care Med.

[41] Permpikul C, Tongyoo S, Viarasilpa T, Trainarongsakul T, Chakorn T, Udompanturak S (2019). Early use of norepinephrine in septic shock resuscitation (CENSER). A randomized trial. Am J Respir Crit Care Med.

[42] Lehman LW, Saeed M, Moody G, Mark R (2010). Hypotension as a risk factor for acute kidney injury in ICU patients. Comput Cardiol.

[43] Asfar P, Meziani F, Hamel JF, Grelon F, Megarbane B, Anguel N (2014). High versus low blood-pressure target in patients with septic shock. N Engl J Med.

[44] Bellomo R, Wan L, May C (2008). Vasoactive drugs and acute kidney injury. Crit Care Med.

[45] Hamzaoui O, Shi R (2020). Early norepinephrine use in septic shock. J Thorac Dis.

[46] Morimatsu H, Singh K, Uchino S, Bellomo R, Hart G (2004). Early and exclusive use of norepinephrine in septic shock. Resuscitation.

[47] Bai X, Yu W, Ji W, Lin Z, Tan S, Duan K (2014). Early versus delayed administration of norepinephrine in patients with septic shock. Crit Care.

[48] Ospina-Tascon GA, Hernandez G, Alvarez I, Calderon-Tapia LE, Manzano-Nunez R, Sanchez-Ortiz AI (2020). Effects of very early start of norepinephrine in patients with septic shock: a propensity score-based analysis. Crit Care.

[49] Immink RV, van den Born BJ, van Montfrans GA, Koopmans RP, Karemaker JM, van Lieshout JJ (2004). Impaired cerebral autoregulation in patients with malignant hypertension. Circulation.

[50] Strandgaard S, Olesen J, Skinhoj E, Lassen NA (1973). Autoregulation of brain circulation in severe arterial hypertension. Br Med J.

[51] Bourgoin A, Leone M, Delmas A, Garnier F, Albanese J, Martin C (2005). Increasing mean arterial pressure in patients with septic shock: effects on oxygen variables and renal function. Crit Care Med.

[52] LeDoux D, Astiz ME, Carpati CM, Rackow EC (2000). Effects of perfusion pressure on tissue perfusion in septic shock. Crit Care Med.

[53] Xu JY, Ma SQ, Pan C, He HL, Cai SX, Hu SL (2015). A high mean arterial pressure target is associated with improved microcirculation in septic shock patients with previous hypertension: a prospective open label study. Crit Care.

[54] Thooft A, Favory R, Salgado DR, Taccone FS, Donadello K, De Backer D (2011). Effects of changes in arterial pressure on organ perfusion during septic shock. Crit Care.

[55] Lee GT, Hwang SY, Jo IJ, Kim TR, Yoon H, Park JH (2019). Associations between mean arterial pressure and 28-day mortality according to the presence of hypertension or previous blood pressure level in critically ill sepsis patients. J Thorac Dis.

[56] Naucler P, Huttner A, van Werkhoven CH, Singer M, Tattevin P, Einav S (2020). Impact of time to antibiotic therapy on clinical outcome in patients with bacterial infections in the emergency department: implications for antimicrobial stewardship. Clin Microbiol Infect.

[57] Peltan ID, Brown SM, Bledsoe JR, Sorensen J, Samore MH, Allen TL (2019). ED door-to-antibiotic time and long-term mortality in sepsis. Chest.

[58] Seok H, Song J, Jeon JH, Choi HK, Choi WS, Moon S (2020). Timing of antibiotics in septic patients: a prospective cohort study. Clin Microbiol Infect.

[59] Seymour CW, Gesten F, Prescott HC, Friedrich ME, Iwashyna TJ, Phillips GS (2017). Time to treatment and mortality during mandated emergency care for sepsis. N Engl J Med.

[60] Singer M (2017). Antibiotics for sepsis: does each hour really count, or is it incestuous amplification?. Am J Respir Crit Care Med.

[61] Sterling SA, Miller WR, Pryor J, Puskarich MA, Jones AE (2015). The impact of timing of antibiotics on outcomes in severe sepsis and septic shock: a systematic review and meta-analysis. Crit Care Med.

[62] Jouffroy R, Vivien B (2020). Implementation of earlier antibiotic administration in patients with severe sepsis and septic shock in Japan: antibiotic action needs time and tissue perfusion to reach target. Crit Care.

[63] Kumar A, Roberts D, Wood KE, Light B, Parrillo JE, Sharma S (2006). Duration of hypotension before initiation of effective antimicrobial therapy is the critical determinant of survival in human septic shock. Crit Care Med.

